# Clinical and radiographic outcomes of revision total knee arthroplasty with a medial pivot design for aseptic failure

**DOI:** 10.1002/jeo2.70847

**Published:** 2026-08-03

**Authors:** Giorgio Cacciola, Francesco Bosco, Federico De Meo, Antongiulio Bruschetta, Claudio Domenico Cobisi, Pietro Cavaliere

**Affiliations:** ^1^ Department of Robotic and Mini‐Invasive Orthopaedic Surgery Humanitas ‘Gradenigo’ Turin Italy; ^2^ Department of Precision Medicine in Medical, Surgical and Critical Care (Me.Pre.C.C.) University of Palermo Palermo Italy; ^3^ Department of Orthopaedics and Traumatology G.F. Ingrassia Hospital Unit Palermo Italy; ^4^ Orthopaedic Institute of Southern Italy “Franco Scalabrino” Messina Italy

**Keywords:** AORI classification, aseptic loosening, bone defects, knee instability, medial pivot, revision total knee arthroplasty

## Abstract

**Purpose:**

The use of medial pivot inserts in revision total knee arthroplasty (rTKA) remains limited, with more constrained designs often preferred. This study aimed to evaluate clinical, radiographic and complication outcomes of rTKA performed with a medial pivot insert for aseptic revision indications in selected patients with preserved ligamentous stability.

**Methods:**

A retrospective analysis was conducted on a prospectively maintained institutional registry. Sixty‐three consecutive patients who underwent first‐time rTKA with a medial pivot revision system between 2017 and 2023 were included. Indications for revision included aseptic loosening, instability, polyethylene wear and arthrofibrosis, in the presence of mild‐to‐moderate bone defects (Anderson Orthopaedic Research Institute [AORI] Type I or IIa) and preserved collateral ligament integrity. Clinical outcomes were assessed using the Knee Society Score (KSS), functional KSS (fKSS) and Western Ontario and McMaster Universities Osteoarthritis Index score. Radiographic evaluation included coronal and sagittal alignment parameters and the presence of radiolucent lines (RLLs). A *p* value < 0.05 was considered statistically significant. Minimum follow‐up was 2 years.

**Results:**

Significant improvements were observed in all clinical outcome measures and knee range of motion (ROM) from preoperative assessment to final follow‐up (all *p* < 0.001). No significant differences in clinical outcomes were detected between patients with AORI Type I and Type IIa bone defects. Radiographic analysis demonstrated satisfactory restoration of coronal and sagittal alignment, with no evidence of component migration or loosening at final follow‐up. RLLs were observed in three cases and were non‐progressive. Two patients (3.2%) required reoperation for periprosthetic joint infection (PJI); no revisions for instability or mechanical failure were recorded.

**Conclusion:**

rTKA performed with a medial pivot insert for aseptic failure resulted in favourable clinical and radiographic outcomes with a low complication rate at short‐ to mid‐term follow‐up. These findings suggest that medial pivot designs may represent a viable option in carefully selected revision cases with mild‐to‐moderate bone loss and preserved ligamentous stability.

**Level of Evidence:**

Level IV.

**Abbreviations**
AORI: Anderson Orthopaedic Research Institute
CR: cruciate‐retaining
DAIR: debridement, antibiotics and implant retention
fKSS: functional Knee Society Score
HKA: hip–knee–ankle angle
KSS: Knee Society Score
LDFA: lateral distal femoral angle
MCID: minimal clinically important difference
MP: medial pivot
MPTA: medial proximal tibial angle
MRSE: methicillin‐resistant Staphylococcus epidermidis
PJI: periprosthetic joint infection
PPF: periprosthetic fracture
PS: posterior‐stabilized
PTS: posterior tibial slope
RLLs: radiolucent lines
ROM: range of motion
ROM_E_
: range of motion, extension
ROM_F_
: range of motion, flexion
rTKA: revision total knee arthroplasty
TKA: total knee arthroplasty
WOMAC: Western Ontario and McMaster Universities Osteoarthritis Index

## INTRODUCTION

Total knee arthroplasty (TKA) is widely regarded as the gold‐standard treatment for end‐stage knee osteoarthritis. Despite excellent long‐term implant survivorship, exceeding 90% at 10 years, up to 10%–20% of patients report persistent dissatisfaction following primary TKA [11, 13, 20]. This proportion further increases after revision total knee arthroplasty (rTKA), where patient‐reported outcomes are generally inferior to those observed after primary procedures [9, 14]. Postoperative dissatisfaction is multifactorial; however, incomplete restoration of native knee biomechanics remains a major challenge affecting functional recovery and patient satisfaction [3, 4].

In recent years, prosthetic designs incorporating a medial pivot (MP) insert have gained increasing attention, as they aim to reproduce more physiological knee kinematics by providing a stable medial compartment with controlled lateral femoral rollback [6]. Several studies have demonstrated improved functional outcomes and higher patient satisfaction with MP designs in primary TKA compared with more traditional implant geometries [6, 12].

Conversely, the use of MP inserts in the revision setting remains limited. In rTKA, surgeons often favour implants with greater constraint to address instability, bone loss or ligament insufficiency [1, 2, 15, 17]. Nevertheless, selected clinical scenarios—such as aseptic loosening or instability in the presence of preserved collateral ligaments—may not require highly constrained designs. In this context, Takagi et al. [16] reported significant improvements in anteroposterior stability and anterior knee pain in patients revised to an MP insert despite well‐positioned posterior‐stabilized (PS) or cruciate‐retaining (CR) implants. Similarly, Vecchini et al. [18] described favourable clinical and radiographic outcomes using a stemmed MP system for aseptic revision, with clinical and radiographic outcomes comparable to those observed after primary MP arthroplasty.

The potential advantages of MP inserts in rTKA include the ability to restore knee kinematics closer to the native joint, enhanced anteroposterior stability due to high medial compartment congruency and a possible reduction in anterior knee pain [5, 7]. However, their indication is generally limited to carefully selected cases with mild‐to‐moderate bone defects (Anderson Orthopaedic Research Institute [AORI] Type I or IIa) and intact collateral ligament structures [8].

The aim of this study was to evaluate clinical outcomes, radiographic alignment and complication rates following rTKA performed with an MP insert for aseptic revision indications. We hypothesized that this design would provide clinically meaningful improvement and stable radiographic fixation in carefully selected patients with mild‐to‐moderate bone defects and preserved ligamentous integrity.

## MATERIALS AND METHODS

### Study design

Following approval from the local Institutional Review Board (Regional Ethics Committee, Calabria Region; approval number 81/2025), a retrospective analysis was conducted using data extracted from a prospectively maintained institutional arthroplasty registry. All consecutive patients who underwent rTKA using the K‐Mod Rev System (Gruppo Bioimpianti) between January 2017 and December 2023 were screened for eligibility.

**Figure 1:**
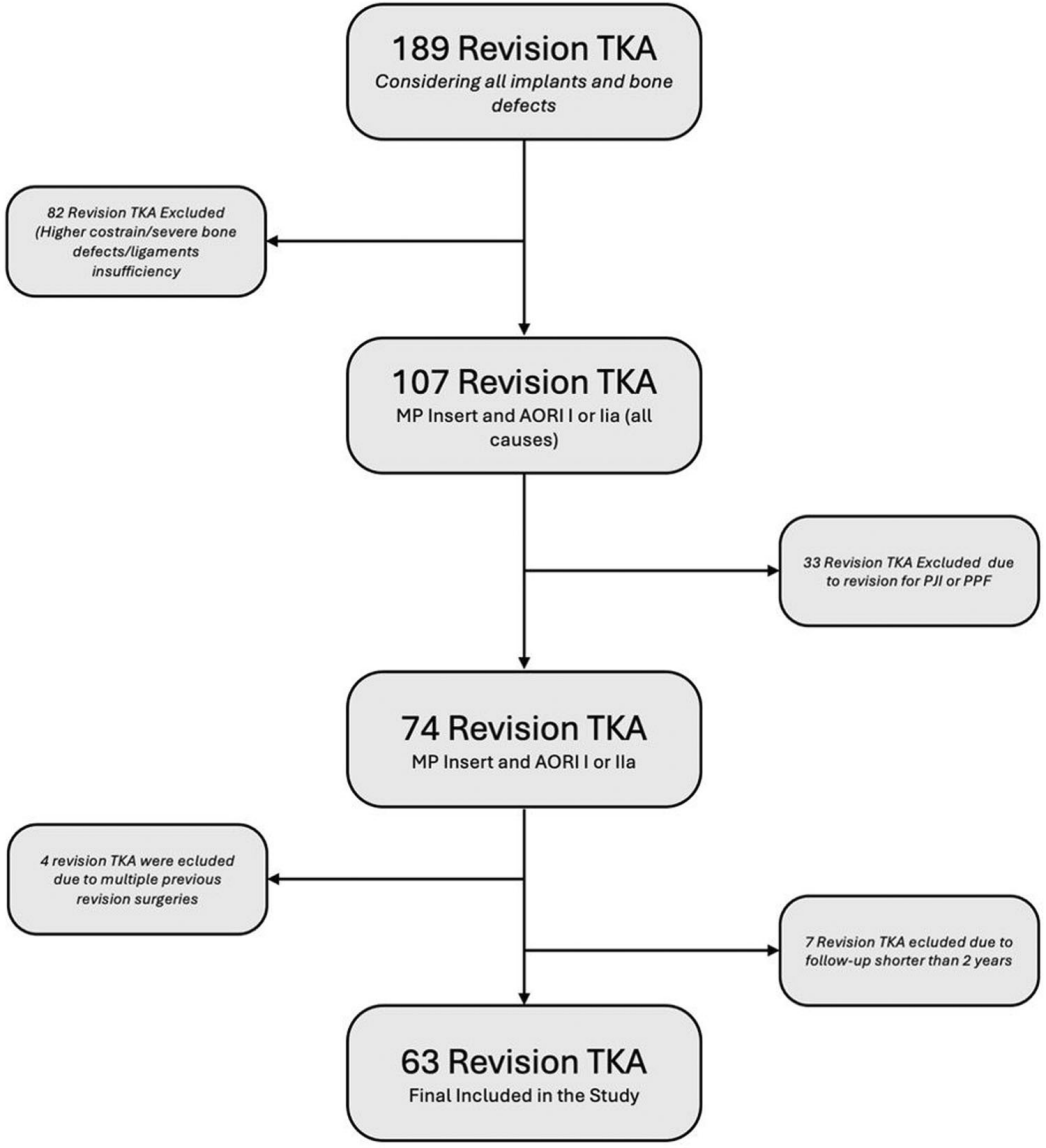
Flow diagram of patient selection. Of 189 revision total knee arthroplasties (rTKAs) assessed, 82 were excluded because they required a higher level of constraint due to severe bone loss or collateral ligament insufficiency. Among the remaining 107 cases with a medial pivot (MP) insert and AORI Type I or IIa defects, 33 were excluded because revision was performed for periprosthetic joint infection (PJI) or periprosthetic fracture (PPF). Of the 74 eligible rTKAs, 11 were further excluded due to multiple prior revision surgeries (*n* = 4) or follow‐up <2 years (*n* = 7). A total of 63 rTKAs were included in the final analysis. AORI, Anderson Orthopaedic Research Institute; TKA, total knee arthroplasty.

Patients were included if they underwent a first‐time revision procedure (defined as a revision following a single previous primary TKA with a CR, PS or medial‐pivot design), received a medial‐pivot insert and had a minimum clinical and radiographic follow‐up of 2 years. Eligible aseptic revision indications included aseptic loosening, instability, polyethylene wear and arthrofibrosis, in the presence of AORI Type I or IIa bone defects and preserved collateral ligament integrity. Exclusion criteria comprised multiple previous revision procedures, the presence of a prior revision implant, use of implants with a different level of constraint and revision performed for periprosthetic joint infection (PJI) or periprosthetic fracture (PPF). During the study period, 189 rTKAs were performed, of which 74 were treated using the K‐Mod Rev System. After application of the inclusion and exclusion criteria, 63 patients (63 rTKAs) were included in the final analysis (Figure 1). Patient demographic and baseline clinical data are reported in Table 1.

**Table 1:** Patient demographics, clinical data and bone defect classification for the 63 rTKAs included in the study.

Data	
Age, years, mean ± SD	76.5 ± 5.9
Gender, women, no. (%)	45 (71.4%)
Body mass index, BMI (kg/m^2^), mean ± SD	29.8 ± 4.9
Number of previous operations, no., mean ± SD	3.2 ± 1.7
Months between previous TKA and revision, mean ± SD	37.8 ± 18.4
Months between revision TKA and last follow‐up, mean ± SD	34.4 ± 9.4
AORI Type I bone defect, no., mean ± SD	37 (58.7%)
AORI Type IIa bone defect, no., mean ± SD	26 (41.3%)

*Note*: Continuous variables are reported as mean ± SD, with ranges provided where applicable, while categorical variables are presented as absolute numbers and percentages.

Abbreviations: AORI, Anderson Orthopaedic Research Institute; BMI, body mass index; rTKAs, revision total knee arthroplasties; SD, standard deviation; TKA, total knee arthroplasty.

The K‐Mod revision implant was selected in cases presenting with mild‐to‐moderate femoral and tibial bone defects (AORI Types I and IIa) and preserved collateral ligament integrity. This modular revision system allows intraoperative customization using stemmed components, offset options, MP inserts and femoral or tibial augments to address a variety of reconstructive scenarios.

### Surgical technique

All revision procedures were performed by the senior author (P. C.) at a single institution, following a standardized perioperative and postoperative protocol. A midline skin incision combined with a medial parapatellar arthrotomy was used in all cases.

After joint exposure, the polyethylene insert was first removed to facilitate access to the femoral and tibial components. The femoral component was carefully explanted using an oscillating saw and thin osteotomes to minimize additional bone loss, followed by removal of the tibial component. Residual cement and fibrous tissue were cleared. Femoral and tibial bone defects were then evaluated intraoperatively and classified according to the AORI classification [8].

Tibial preparation was performed first using an extramedullary alignment guide. The tibial cut was adjusted to restore appropriate joint line height and coronal alignment, and tibial augments were applied when necessary to compensate for bone defects. Stemmed tibial components were used in all cases to enhance fixation and load transfer. Femoral preparation was subsequently carried out using an intramedullary alignment guide, with distal and posterior femoral augments employed as required to address bone loss and restore femoral geometry.

Soft‐tissue balancing was performed to achieve symmetrical, rectangular extension and flexion gaps. Attention was paid to restoring stability throughout the full range of motion (ROM), including mid‐flexion. Trial components were inserted to assess knee kinematics, ROM and anteroposterior as well as coronal stability. The integrity of the collateral ligaments was systematically evaluated to confirm suitability for an MP insert and to exclude the need for higher levels of constraint.

Final component implantation was performed once satisfactory alignment, gap balance and stability were achieved. The MP insert was selected to provide functional anteroposterior stability while preserving near‐physiological knee kinematics. All components were implanted according to the manufacturer's recommendations.

### Clinical outcomes

Clinical evaluation was performed preoperatively and at 6 months, 1 year and annually thereafter using the Knee Society Score (KSS), functional Knee Society Score (fKSS) and the Western Ontario and McMaster Universities Osteoarthritis Index (WOMAC). At each follow‐up visit, knee ROM was assessed and recorded as maximum active flexion (ROM_F_) and maximum active extension (ROM_E_). All postoperative complications, reoperations and revision procedures were prospectively documented.

### Radiographic outcomes

Standardized anteroposterior and lateral weight‐bearing knee radiographs, as well as full‐length standing lower limb radiographs, were obtained preoperatively, at 3 and 6 months postoperatively, at 1 year and annually thereafter until final follow‐up. All radiographic images were digitally acquired and analysed using HOROS DICOM Viewer (Horos Project, macOS).

Radiographic parameters included the hip–knee–ankle angle (HKA; varus alignment expressed as negative values), lateral distal femoral angle (LDFA; values < 90° indicating valgus alignment), medial proximal tibial angle (MPTA; values < 90° indicating varus alignment) and posterior tibial slope (PTS). The presence and distribution of radiolucent lines (RLLs) were assessed on both anteroposterior and lateral views according to the Knee Society radiographic evaluation system.

### Statistical analysis

Statistical analysis was performed using SPSS software (Version 31.1; IBM Corp.). Continuous variables are presented as mean ± standard deviation, whereas categorical variables are reported as absolute frequencies and percentages. Normality of data distribution was assessed using the Shapiro–Wilk test. Clinical outcome scores (KSS, functional KSS, WOMAC, ROMF and ROME) were analysed as continuous variables using parametric or non‐parametric tests according to data distribution. Paired comparisons between preoperative and postoperative values were performed using paired Student's *t* tests for normally distributed variables and Wilcoxon signed‐rank tests for non‐normally distributed variables. Comparisons between patients with AORI Type I and Type IIa bone defects were conducted using independent Student's *t* tests or Mann–Whitney *U* tests, as appropriate. Longitudinal changes across follow‐up time points were analyzed using repeated‐measures analysis of variance (ANOVA) for normally distributed variables and Friedman tests for non‐parametric variables. Post hoc pairwise comparisons were adjusted using Bonferroni correction when appropriate. Radiographic outcomes were analyzed descriptively, and RLLs were reported as a dichotomous variable. Minimal clinically important difference (MCID) thresholds were defined a priori based on prior arthroplasty literature as 10–12 points for WOMAC, 5–7 points for the KSS and 6–9 points for the functional KSS. Observed changes were interpreted considering both statistical significance and clinical relevance. No a priori sample size calculation was performed due to the retrospective design of the study; however, a post hoc power analysis was conducted to verify that the available sample size was adequate to detect differences exceeding MCID thresholds. Post hoc analysis demonstrated greater than 90% power for detecting clinically meaningful improvements in the primary outcome measures. A *p* value < 0.05 was considered statistically significant for all analyses.

## RESULTS

Patient demographic and baseline clinical characteristics are summarized in Table 1. The mean age at the time of revision surgery was 76.5 ± 5.9 years, and the cohort included 45 women (71.4%). The mean interval between primary TKA and rTKA was 37.8 ± 18.4 months, and the mean follow‐up duration was 34.4 ± 13.4 months.

Aseptic loosening was the most common indication for revision, accounting for 49 cases (77.8%), followed by instability in 6 cases (9.5%). Polyethylene wear and arthrofibrosis were each observed in 4 cases (6.3%). According to the AORI classification, femoral and tibial bone defects were categorized as Type I in 37 cases (58.7%) and Type IIa in 26 cases (41.3%).

### Complications

Reoperations were required in two patients (3.2%) due to PJI. One early infection occurred 2 weeks after the index revision procedure and was managed with debridement, antibiotics and implant retention (DAIR). Intraoperative cultures from five periprosthetic tissue samples isolated methicillin‐sensitive Staphylococcus epidermidis. The patient received intravenous therapy followed by oral antibiotics for a total of 8 weeks, with no evidence of persistent infection at subsequent follow‐up. The second case presented 6 months postoperatively with a draining sinus tract. Joint aspiration demonstrated a synovial white blood cell count of 55,000 cells/µL with 94% polymorphonuclear leukocytes, and cultures isolated methicillin‐resistant Staphylococcus epidermidis. A two‐stage revision was performed, consisting of component removal and implantation of an antibiotic‐loaded cement spacer, followed by 8 weeks of targeted antibiotic therapy. Reimplantation was performed 2 months after resolution of clinical symptoms and normalization of inflammatory markers. No additional complications were recorded during follow‐up.

### Clinical outcomes

All clinical outcome measures, including the WOMAC, KSS and functional KSS, demonstrated significant improvement from the preoperative assessment to the final follow‐up (all *p* < 0.001). The magnitude of improvement substantially exceeded established MCID thresholds, with mean WOMAC improving by 34 points, KSS by 40 points and functional KSS by 35 points, confirming that the observed benefits were not only statistically significant but also clinically meaningful. Similarly, knee ROM showed significant postoperative improvement, with a reduction in extension deficit and an increase in maximum flexion compared with preoperative values.

At final follow‐up, no statistically significant differences in clinical or functional outcomes were observed between patients with AORI Type I and Type IIa bone defects (Table 2), indicating that mild‐to‐moderate bone loss did not adversely affect postoperative recovery within the indications of the MP revision system.

**Table 2:** Clinical outcomes before revision surgery and at final follow‐up.

	Total (63 rTKA, 100%)	AORI I (37 rTKA, 58.7%)	AORI IIa (26, 41.3%)	*p* Value
WOMAC score				
Preop.	62.7 ± 8.4	62.4 ± 8.2	63.1 ± 8.5	0.745
3 months	35.4 ± 5.3	34.9 ± 5.7	35.6 ± 6.1	0.647
6 months	35.1 ± 5.2	34.5 ± 5.5	36.1 ± 5.4	0.256
1 year	29.8 ± 4.3	29.3 ± 4.8	30.4 ± 4.7	0.369
2 years	28.7 ± 4.5	28.5 ± 5.1	29.3 ± 4.3	0.504
*p* Value	<0.001	<0.001	<0.001	—
KSS				
Preop.	45.4 ± 9.2	45.7 ± 8.8	45.9 ± 9.1	0.931
3 months	71.2 ± 10.2	72 ± 9.9	69.9 ± 9.8	0.40
6 months	74.3 ± 11.1	74.1 ± 10.2	74.5 ± 9.9	0.877
1 year	85.6 ± 9.8	85.9 ± 9.4	85.2 ± 8.7	0.762
2 years	85.8 ± 9.4	86.1 ± 8.2	85.4 ± 7.9	0.735
*p* Value	<0.001	<0.001	<0.001	—
Functional KSS				
Preop.	39.4 ± 13.2	40.2 ± 11.2	38.4 ± 12.2	0.554
3 months	39.4 ± 13.2	40.2 ± 11.2	38.4 ± 12.2	0.554
6 months	63.4 ± 7.9	64.2 ± 8.1	62.9 ± 8.6	0.548
1 year	74.2 ± 8.4	74.9 ± 9.4	73.8 ± 9.2	0.645
2 years	74.8 ± 8.7	75.2 ± 9.1	74.3 ± 9.1	0.701
*p* Value	<0.001	<0.001	<0.001	—
ROM_E_ (°)				
Preop.	9.1 ± 5.8	9 ± 6.1	8.9 ± 5.5	0.944
3 months	5.8 ± 4.6	5.5 ± 6.3	5.3 ± 5.1	0.883
6 months	3.5 ± 5.3	3.4 ± 5.8	3.6 ± 5.4	0.901
1 year	3.4 ± 5.4	3.3 ± 5.5	3.6 ± 6.1	0.828
2 years	3.4 ± 5.5	3.2 ± 4.8	3.6 ± 5.3	0.772
ROM_f_ (°)				
Preop.	93.2 ± 11.4	91.9 ± 13.4	94.8 ± 11.7	0.356
3 months	104.5 ± 8.9	105.6 ± 11.9	103.9 ± 10.7	0.642
6 months	116.5 ± 12.4	118.9 ± 11.5	114.8 ± 10.9	0.202
1 year	116.9 ± 11.9	119 ± 10.9	115.9 ± 10.4	0.298
2 years	116.8 ± 11.5	119.2 ± 11.4	116.2 ± 10.8	0.324
*p* Value	<0.001	<0.001	<0.001	—

*Note*: Functional scores and ROM are reported preoperatively and postoperatively to assess clinical improvement following rTKA. Data are presented as mean ± standard deviation.

Abbreviations: AORI, Anderson Orthopaedic Research Institute; KSS, Knee Society Score; ROM, range of motion; rTKA, revision total knee arthroplasty; WOMAC, Western Ontario and McMaster Universities Osteoarthritis Index.

### Radiographic outcomes

Postoperative radiographic analysis demonstrated satisfactory restoration of both coronal and sagittal limb alignment following rTKA. In the overall cohort, the HKA angle improved toward neutral alignment, decreasing from a preoperative mean of 6.2 ± 3.9° to 1.8 ± 1.6° postoperatively. The LDFA and MPTA similarly showed correction toward neutral alignment. PTS demonstrated a modest reduction following revision surgery.

**Figure 2:**
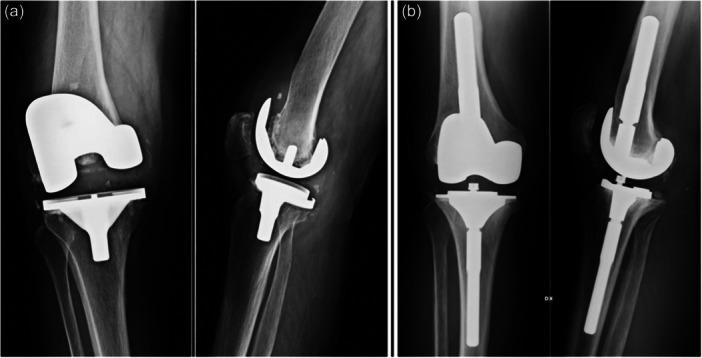
Preoperative and postoperative radiographic evaluation of rTKA in a 63 year old men. Anteroposterior and lateral radiographs of the right knee showing (a) preoperative failure due to aseptic loosening of the femoral component associated with an AORI type IIa bone defect and (b) postoperative appearance after rTKA with the use of K‐Mod revision medial pivot implant. Postoperative images demonstrate restoration of limb alignment and implantation of revision components with femoral and tibial stems, providing stable fixation and appropriate alignment. No radiolucent lines are visible at the 4‐year radiographic follow‐up. AORI, Anderson Orthopaedic Research Institute; rTKA, revision total knee arthroplasty.

At final follow‐up, postoperative radiographic parameters were comparable between the AORI Type I and Type IIa groups, indicating similar alignment accuracy irrespective of bone defect severity (Figure 2; Table 3).

**Table 3:** Preoperative and postoperative coronal and sagittal alignment parameters for the overall cohort and stratified by AORI bone defect Type (I and IIa).

	Total (63 rTKA, 100%)	AORI I (37 rTKA, 58.7%)	AORI IIa (26, 41.3%)	*p* Value
HKA (°)				
Preoperative	6.2 ± 3.9	6.0 ± 3.7	6.5 ± 4.2	0.61
Postoperative	1.8 ± 1.6	1.7 ± 1.5	1.9 ± 1.7	0.58
LDFA (°)				
Preoperative	86.8 ± 3.6	87.0 ± 3.4	86.5 ± 3.8	0.64
Postoperative	89.2 ± 2.0	89.3 ± 1.9	89.0 ± 2.2	0.60
MPTA (°)				
Preoperative	85.6 ± 4.1	85.8 ± 3.9	85.3 ± 4.4	0.67
Postoperative	88.8 ± 2.3	89.0 ± 2.1	88.5 ± 2.6	0.52
PTS (°)				
Preoperative	6.4 ± 2.7	6.5 ± 2.6	6.3 ± 2.9	0.78
Postoperative	5.0 ± 1.7	4.9 ± 1.6	5.1 ± 1.8	0.71

*Note*: Hip–knee–ankle angle (HKA), lateral distal femoral angle (LDFA), medial proximal tibial angle (MPTA) and posterior tibial slope (PTS) are presented as mean ± standard deviation. No statistically significant differences were detected between AORI I and AORI IIa groups at either time point.

Abbreviations: AORI, Anderson Orthopaedic Research Institute; rTKA, revision total knee arthroplasty.

RLLs were observed in three cases, predominantly involving isolated tibial zones (Zones 1 and 4 according to the Knee Society radiographic evaluation system). These RLLs were non‐progressive over time and were not associated with clinical symptoms. No radiographic evidence of component migration or loosening was detected, and all femoral and tibial components appeared stable at the most recent radiographic evaluation.

## DISCUSSION

The present study evaluated the clinical, radiographic and complication outcomes of rTKA performed with an MP design in a selected cohort of patients with aseptic revision indications, mild‐to‐moderate bone defects and preserved collateral ligament integrity. The most important finding of the present study is that rTKA performed with an MP insert provided clinically meaningful improvement while maintaining radiographic stability and a low complication profile at short‐ to mid‐term follow‐up. These findings are particularly relevant given the ongoing debate regarding the optimal level of constraint in rTKA. Notably, outcomes were comparable between patients with AORI type I and type IIa bone defects, suggesting that MP designs may represent a viable option in carefully selected revision cases characterized by mild‐to‐moderate bone loss and preserved ligamentous stability.

The observed improvements in WOMAC, KSS and fKSS are consistent with the limited available literature on MP implants in the revision setting. Importantly, the magnitude of these improvements exceeded widely accepted MCID thresholds, reinforcing the clinical relevance of the findings. Takagi et al. [16] reported a significant reduction in anteroposterior instability symptoms and anterior knee pain following revision to an MP insert in patients with persistent instability despite well‐positioned CR or PS implants. Similarly, Vecchini et al. [18] demonstrated favourable clinical and radiographic outcomes using a stemmed MP revision system for aseptic loosening, with implant survivorship comparable to that observed after primary MP arthroplasty. Nevertheless, evidence regarding the role of MP designs in rTKA remains scarce. In this context, the present study contributes additional data to an underreported field by analysing a homogeneous cohort of revision procedures performed for aseptic loosening or instability and stratifying outcomes by bone defect severity [11, 13, 20].

Radiographic analysis demonstrated satisfactory restoration of both coronal and sagittal alignment, with no significant differences observed between AORI Type I and Type IIa defects. These findings suggest that accurate limb alignment can be reliably achieved with an MP revision system even in the presence of mild‐to‐moderate bone loss, provided that appropriate reconstruction strategies—including the use of stems and augments—are adopted [8, 17]. RLLs were detected in a limited number of cases, were non‐progressive and showed no association with clinical symptoms. Importantly, no evidence of component migration or aseptic loosening was identified at final follow‐up, supporting the mechanical stability of the implant when used within properly selected indications. Similar radiographic stability has been reported in prior studies evaluating stemmed revision constructs, highlighting the importance of achieving secure fixation in revision arthroplasty [18]. From a biomechanical perspective, restoration of neutral or near‐neutral alignment remains a key determinant of load distribution and implant longevity in rTKA. Malalignment has been consistently associated with uneven load transfer and increased risk of early mechanical failure, particularly in the revision setting. Achieving stable fixation while avoiding excessive constraint is particularly relevant in this setting, as higher constraint levels have been associated with increased stresses at the bone–implant interface and a potential risk of mechanical failure [10, 17]. The present radiographic findings suggest that an MP design can provide adequate intrinsic stability in selected revision scenarios, although higher levels of constraint may still be required in more complex cases.

The overall complication rate observed in this series was low and comparable to previously reported rates following rTKA. Two cases of PJI were recorded, one successfully managed with DAIR and one requiring a two‐stage revision procedure. Notably, no revisions for instability or mechanical failure were observed, reinforcing the concept that MP inserts can provide adequate stability in revision settings when collateral ligament integrity is preserved. These findings are in line with those reported by Vecchini et al. [18], who described a similar reoperation rate, including one late failure due to aseptic loosening requiring conversion to a hinged implant and two cases of deep PJI.

From a biomechanical standpoint, the MP concept is designed to replicate native knee kinematics by creating a highly congruent medial compartment that serves as a stable pivot during flexion, while allowing controlled lateral femoral rollback. In the revision setting, this design may provide adequate intrinsic stability in selected patients with preserved ligamentous integrity and mild‐to‐moderate bone loss [10, 19]. This approach may be particularly suitable in cases characterized by limited bone loss and instability in the absence of major ligament insufficiency, where restoration of near‐physiological kinematics may represent a reasonable strategy in carefully selected revision scenarios.

Several limitations of this study should be acknowledged. First, the retrospective design inherently limits the ability to establish causal relationships and introduces the potential for selection bias. However, data were derived from a prospectively maintained institutional registry, and all procedures were performed using a standardized surgical technique and perioperative protocol, partially mitigating this limitation. Nevertheless, all procedures were performed by a single experienced surgeon at a single institution, which may limit the external validity and generalizability of the present findings to different surgical settings. Second, the absence of a control group treated with more constrained revision implants precludes direct comparison between different implant philosophies. The primary objective of this study, however, was not to demonstrate superiority over alternative designs but rather to evaluate the clinical performance of an MP system in a carefully selected revision population. Third, although the sample size was moderate, post‐hoc power analysis confirmed adequate statistical power to detect clinically meaningful differences exceeding established MCID thresholds for the primary outcome measures. Fourth, the follow‐up duration reflects short‐ to mid‐term results and does not allow conclusions regarding long‐term implant survivorship or late mechanical failures. Future prospective studies with longer follow‐up are warranted to confirm the durability of these findings. Finally, the study population was intentionally restricted to patients with mild‐to‐moderate bone defects and preserved collateral ligament integrity. Therefore, the present results should not be generalized to more complex revision scenarios requiring higher levels of constraint. Furthermore, the strict selection criteria adopted in the present study inevitably introduced a degree of selection bias, as only patients with AORI Type I or IIa bone defects and preserved collateral ligament integrity were considered eligible for an MP revision system.

## CONCLUSIONS

rTKA performed with an MP insert for aseptic loosening or instability was associated with statistically significant and clinically meaningful improvement, satisfactory restoration of limb alignment and a low complication rate at short‐ to mid‐term follow‐up. Comparable outcomes were observed between patients with AORI type I and type IIa bone defects, suggesting that this implant design may be considered in selected revision cases with mild‐to‐moderate bone loss and preserved ligamentous stability. When appropriately indicated, MP revision implants may represent a viable option in carefully selected revision cases with preserved ligamentous stability and mild‐to‐moderate bone loss. Further prospective, comparative studies with longer follow‐up are needed to confirm these findings and to better define the role of MP designs in rTKA.

## AUTHOR CONTRIBUTIONS

Giorgio Cacciola and Pietro Cavaliere contributed to the conceptualization and design of the study. Giorgio Cacciola was responsible for data collection. Francesco Bosco supervised the project. Antongiulio Bruschetta, Federico De Meo and Claudio Domenico Cobisi contributed to data interpretation and manuscript preparation. All authors reviewed and approved the final version of the manuscript.

## CONFLICT OF INTEREST STATEMENT

Pietro cavaliere is a paid Consultant by Gruppo Bioimpianti (Peschiera Borromeo, Italy).

## ETHICS STATEMENT

This study was approved by the Institutional Review Board (Regional Ethics Committee, Calabria Region; approval number 81/2025). All patients provided informed consent for the use of their clinical data for research and publication purposes, in accordance with institutional and ethical standards.

## Data Availability

The data that support the findings of this study are available on request from the corresponding author. The data are not publicly available due to privacy or ethical restrictions.
